# Simulation-based medical education in Thailand: a cross-sectional online national survey

**DOI:** 10.1186/s12909-022-03369-9

**Published:** 2022-04-20

**Authors:** Polpun Boonmak, Suwannee Suraseranivongse, Ngamjit Pattaravit, Suhattaya Boonmak, Tachawan Jirativanont, Tripop Lertbunnaphong, Rajin Arora, Jittiya Watcharotayangul, Intanon Imsuwan, Panithan Kwangwaropas, Borwon Wittayachamnankul

**Affiliations:** 1 Department of Anesthesiology, Faculty of Medicine, Khon Kaen University, Khon Kaen, 40002 Thailand; 2 Department of Anesthesiology, Faculty of Medicine Siriraj Hospital, Mahidol University, Bangkok, 10700 Thailand; 3 Department of Anesthesiology, Faculty of Medicine, Prince of Songkla University, Songkla, 90110 Thailand; 4 Department of Obstetrics and Gynecology, Faculty of Medicine Siriraj Hospital, Mahidol University, Bangkok, 10700 Thailand; 5 Chulabhorn Royal Academy, Bangkok, 10210 Thailand; 6 Department of Anesthesiology, Faculty of Medicine Ramathibodi Hospital, Mahidol University, Bangkok, 10400 Thailand; 7 Department of Emergency Medicine, Faculty of Medicine, Thammasat University, Pathumthani, 12120 Thailand; 8 Simulation Center for Military Medicine, Phramongkutklao College of Medicine, Bangkok, 10700 Thailand; 9 Department of Emergency Medicine, Faculty of Medicine, Chiangmai University, Chiangmai, 50200 Thailand

**Keywords:** Medical education, Simulation training, Thailand

## Abstract

**Background:**

Simulation-Based Medical Education (SBME) is a teaching method commonly used in undergraduate medical education. Although Thai medical schools have developed a system that incorporates SBME, various aspects of that system require improvement. We surveyed medical school administrators, instructors, and students about SBME in their institutions and the obstacles involved in its implementation, as well as their experiences, expectations, and attitudes regarding the current system.

**Methods:**

We conducted a cross-sectional online survey between August 2019 and July 2020 among administrators, instructors, and 6th-year medical students. A structured questionnaire was developed and distributed to volunteers as an online survey. We recorded details about the SBME system as well as participant characteristics, obstacles, experiences, expectations, and attitudes. We used descriptive statistics as appropriate.

**Results:**

We received responses from 15 (68.2%) administrators, 186 instructors, and 371 (13.7%) sixth-year medical students. SBME was commonly used in teaching and evaluation but less so in research. It was mainly used to improve psychomotor tasks, knowledge, patient care, and communication skills. The expected outcomes were improvements in students’ performance, knowledge, and practice. The clinical courses were longer and had fewer participants than the pre-clinical courses. Obstacles encountered included shortages of faculty and simulators, time and space limitations, inadequate faculty training, and insufficient financial support. The administrators surveyed had positive attitudes toward SBME. Medical students reported having experience with SBME and strongly agreed that it was beneficial; however, they expected fewer students per class and more learning time to be devoted to these methods.

**Conclusions:**

SBME in Thailand is focused on teaching and assessment. The system could be improved through better-trained faculty, greater available space, more simulators, and sufficient funding. There were also some aspects that failed to meet students’ expectations and need to be addressed. However, participants expressed positive attitudes toward SBME.

**Trial Registration:**

TCTR20210524003 (Thai Clinical Trials Registry).

**Supplementary Information:**

The online version contains supplementary material available at 10.1186/s12909-022-03369-9.

## Introduction

Simulation-Based Medical Education (SBME) is a training and assessment tool commonly used in undergraduate medical education. SBME teaching methods often incorporate training with part-task trainers (a training device that is designed for the education of only a particular task), high fidelity mannequins (a manikin closely resembles human anatomy and can reproduce or mimic human physiology), standardized patients (the individuals who are specially trained to act as patients for the instruction, practice, and assessment of medical examination skills), screen-based simulations (the form of simulation in which a clinical scenario with one or more patients is presented through a digital screen surface), and cadavers. This allows medical students to practice their skills safely in simulated environments before performing procedures on patients. As such, it plays a critical role in improving the education of healthcare professionals as well as the quality of care. SBME has been shown to improve knowledge, technical skills, and non-technical skills such as teamwork, decision-making, situation awareness, and communication significantly more effectively than in non-SBME education [1–5].

Over the past decade, SBME in Thai medical curricula has increased in interest and demand as an education strategy. Advantages of SBME, such as experiential learning, deliberate practice, transformative learning, and debriefing technique, could deliver comprehensive skills required to provide safe and effective patient care. It also is an effective educational strategy to reduce performance gaps and medical errors [3–5]. Medical schools have implemented SBME in various ways, such as integrating it into their curricula and developing instructor and assistant training programs. In addition, many schools provide space and funding for equipment such as mannequins. However, limits with regard to funding and personnel remain [6]. We thus collaborated with the Thai Society for Simulation in Healthcare (ThaiSSH) in conducting a survey to better understand the current state of SBME in Thailand. Administrators, instructors, and students at Thai medical schools were asked about SBME at their institution, obstacles they have encountered, and their experiences, expectations, and attitudes regarding the current system. Such data will be help policymakers and faculty in medical schools develop strategies to improve SBME in the country.

## Methods

### Study design

This prospective descriptive study was based on a cross-sectional online survey conducted in Thailand between August 1, 2019 and July 31, 2020. We obtained Institute Review Board (IRB) approval from Khon Kaen University before the commencement of the study. We designed and developed a structured questionnaire based on those used in previous studies and adjusted it for use in a Thai context [7–9]. We had six experts (one expert in questionnaire development) in the content area to test the content validity. After receiving the responses from experts, we edited each item until accepted as highly relevant by experts. We then conducted a pilot study to determine its reliability (30 instructors, 30 sixth-year medical students, and five academic administrators) and made the necessary modifications based on the results. Reliability was assessed using Cronbach’s alpha coefficient and determined to be acceptable (0.79 for the instructor questionnaire and 0.85 for the student questionnaire). Data were reported in accordance with STrengthening the Reporting of OBservational Studies in Epidemiology (STROBE) guidelines.

### Questionnaires

Participants filled out an electronic consent form before taking the questionnaire. There were three separate questionnaires for administrators, instructors, and medical students. The administrator questionnaire consisted of two sections: participant characteristics (name, position, school name, phone number, and e-mail) and SBME learning objectives, obstacles, and attitudes. The instructor questionnaire consisted of two sections: participant characteristics (age, school name, department, and e-mail) and SBME implementation and obstacles (consisting of questions to determine the expected outcomes, types of simulators, teaching characteristics, topics taught, and obstacles). The student questionnaire consisted of two sections: participant characteristics (age, gender, medical school name) and SBME objectives and implementation (consisting of questions to determine learning objectives, types of simulators, experiences, expectations, satisfaction with regard to essential procedural skill training, and attitude). A five-point Likert rating scale was used to measure administrators’ and medical students’ attitudes regarding SBME (1 = strongly disagree; 2 = disagree; 3 = neither agree nor disagree; 4 = agree; 5 = strongly agree). The electronic questionnaire took about 20 min to complete.

### Data collection

We defined administrators as those assigned responsibilities for undergraduate programs or SBME by the dean of their school (deputy dean for academic affairs or head of the simulation center). We defined instructors as those who taught at medical schools during the study period. Students included were in their sixth year of medical school during the study period. In Thailand, medical students spend three years on a pre-clinical study and three years on a clinical study. The sixth-year is the final year of their medical curriculum. Volunteers were recruited using an online survey (Google Forms, Google Inc.). The first page of each questionnaire described the purpose of the study and information about participation (including the right to withdraw). Only after agreeing to participate could potential respondents continue to the online survey. We distributed the administrator questionnaire to the administrators of 22 medical schools and asked that they be forwarded to the person responsible for undergraduate training at each school. We distributed the instructor and student questionnaires to a contact person at each medical school (22 medical schools) to distribute through their official e-mail list. In 2020, there was a total of 2,715 6th-year medical students enrolled nationally. The authors also distributed each questionnaire through the ThaiSSH website. In addition, we sent out a reminder to our contacts to encourage them to fill out our survey.

### Statistical analysis

Data analysis was performed using IBM SPSS Statistics for Macintosh, Version 26.0 (IBM Corp, Armonk, NY). We used descriptive statistics to describe participant characteristics. Categorical data were presented as percentage and frequency and continuous data as mean and standard deviation. We calculated the proportion using the number of participants with non-missing data. We compared SBME learning experiences and expectations in each subject using two proportion Z tests and calculated a difference in proportion with a 95% confidence interval or *p*-value, as appropriate. The estimated required sample size out of the 2,715 enrolled medical students was determined to be 337 based on a 95% confidence interval and margin of error of 5%.

## Results

### Participant characteristics

We received responses from 15 administrators (68.2% of all administrators) from 15 medical schools. All of whom were clinicians responsible for simulation training. We received responses from 186 instructors from 16 medical schools, 154 of whom (82.8%) used SBME in their classes. Those who used SBME taught microbiology (*n* = 2), anatomy (*n* = 3), otorhinolaryngology (*n* = 3), radiology and rehabilitation (*n* = 3), ophthalmology (*n* = 6), forensic medicine (*n* = 7), psychiatry (*n* = 9), orthopedics (*n* = 10), surgery (*n* = 11), internal medicine (*n* = 16), obstetrics and gynecology (*n* = 17), pediatrics (*n* = 19), anesthesiology (*n* = 22), and emergency medicine (*n* = 23). The mean (SD) age was 40.4 (8.4) years. Because we were not able to accurately determine the total number of instructors who received a link to the questionnaire, it was not possible to calculate the response rate. We received responses from 371 6th-year medical students (13.7% of all 6th-year medical students in 2020) from 12 medical schools. We excluded four students (1.1%) who reported no experience with SBME, leaving 367 to be included in the analysis. The mean (SD) age was 23.9 (1.0) years old, and 53.1% were female.

### Objectives, outcomes, and implementation of SBME in Thai medical schools

Table 1 details the objectives, outcomes, and characteristics of SBME in Thai medical schools. All administrators reported that SBME was used in their curriculum, mainly to improve psychomotor tasks, medical knowledge, patient care, and communication skills. SBME was more commonly used in teaching and evaluation than in research. More instructors reported that they expected SBME to improve students’ performance than their knowledge or practice. Furthermore, few expected SBME to improve students’ attitudes or patient outcomes. Instructors mainly used part-task trainers, following by high-fidelity mannequins and standardized patients. A minority also used screen-based simulations and cadavers. The duration of SBME instruction was longer for clinical year medical students than for those in their pre-clinical years. However, that of SBME preparation was longer for pre-clinical students. Instructors taught more courses per year for clinical students than pre-clinical students, but the latter had a greater average number of students per class. Administrators reported that SBME is essential in the medical curriculum, needs to be integrated into the medical curriculum, and should be required in medical education, with mean Likert scores of 4.53 (0.52), 4.53 (0.52), and 4.53 (0.52), respectively.

**Table 1:** Objectives, outcomes, and implementation of SBME in Thai medical schools

Topic				
**Administrator data (*N* = 15)**				
**Learning objective (*n* (%))**	**Overall**	**For teaching**	**For evaluation**	**For research**
Psychomotor tasks	15 (100)	15 (100)	11 (73.3)	5 (33.3)
Medical knowledge	14 (93.3)	14 (93.3)	13 (86.7)	4 (26.7)
Patient care	14 (93.3)	14 (93.3)	13 (86.7)	4 (26.7)
Communication skills	14 (93.3)	12 (80.0)	12 (80.0)	4 (26.7)
Professionalism	12 (80.0)	10 (66.7)	10 (66.7)	4 (26.7)
Decision making	12 (80.0)	11 (73.3)	10 (66.7)	3 (20.0)
Team management	12 (80.0)	12 (80.0)	11 (73.3)	3 (20.0)
Leadership	11 (73.3)	9 (60.0)	8 (53.3)	2 (13.3)
**Instructor data (*N* = 154) **^**a**^	**Pre-clinical year (1**^**st**^**-3**^**rd**^** year; *N* = 26)**		**Clinical year (4**^**th**^**-6**^**th**^** year; *N *= 133)**	
**The expected outcome of the SBME course (*n* (%))**				
Improving knowledge	17 (65.4)		71 (53.4)	
Improving attitude	14 (53.8)		61 (45.9)	
Improving performance	21 (80.8)		106 (79.7)	
Improving practice	16 (61.5)		86 (64.7)	
Improving patient outcomes	10 (38.5)		43 (32.3)	
**Type of simulators (*n* (%))**				
Part task trainer	21 (80.8)		98 (73.7)	
High fidelity mannequin	7 (26.9)		74 (55.6)	
Standardized patients	16 (61.5)		68 (51.1)	
Screen-based simulation	4 (15.4)		15 (11.3)	
Cadaver	4 (15.4)		6 (4.5)	
**Teaching characteristics (mean (SD))**				
Teaching duration per course (hours)	2.7 (0.7)		3.3 (4.6)	
Preparation duration (hours)	4.5 (3.2)		3.0 (5.1)	
Courses per year (times)	2.8 (3.0)		6.7 (4.7)	
Students per class (person)	33.0 (22.7)		15.4 (9.1)	

^a^Five instructors taught both pre-clinical and clinical year medical students

### Obstacles to SBME implementation in Thai medical schools

Table 2 shows the obstacles encountered with regard to SBME in medical schools. Many administrators reported having no simulation center (6 out of 15 schools), no administration system (9 out of 15 schools), or insufficient financial support (5 out of 15 schools). In addition, many reported insufficient space for SBME, with the main problem being a lack of controlled rooms and observation rooms. Many also indicated shortages of simulators (especially screen-based simulators) and high-fidelity mannequins. The most common obstacle reported was insufficient SBME personnel including simulation technicians, researchers, standardized patient trainers, and research assistants. Instructors reported similar issues, with insufficient personnel, time, financial support, simulators, and space being the most commonly cited.

**Table 2:** Obstacles to effective SBME implementation in Thai medical schools

Topic			
**Administrator data (*N* = 15; *n* (%))**			
**Lack of administration system**			
No administration system	9 (60.0)	No simulation center	6 (40.0)
Insufficient financial support	5 (33.3)		
**Space shortages**			
Controlled room	9 (60.0)	Observation room	8 (53.3)
Lecture room	7 (46.7)	Training room	7 (46.7)
Debrief room	6 (40.0)	Storage room	6 (40.0)
Office room	6 (40.0)		
**Simulator shortages**			
Screen-based simulation	12 (80.0)	High fidelity mannequins	10 (66.7)
Cadaver	7 (46.7)	Standardized patients	6 (40.0)
Part task trainer	5 (33.3)		
**Faculty shortages**			
Simulation technicians	14 (93.3)	Researchers	14 (93.3)
Standardized patient trainers	13 (86.7)	Researcher assistants	13 (86.7)
Course directors	10 (66.7)	Officers	10 (66.7)
Educators	10 (66.7)	Instructors	7 (46.7)
**Instructor data (*N* = 154; *n* (%))**			
Insufficient faculty	115 (74.4)	Shortage of simulators	107 (69.5)
Insufficient time	97 (63.0)	Insufficient faculty training	91 (59.1)
Insufficient financial support	88 (57.1)	Shortage of space	73 (47.4)

### SBME objectives, expectations, and implementation among Thai medical students

Table 3 shows the objectives and implementation of SBME reported by Thai medical students. Medical students reported having experience with SBME in learning clinical procedures, physical examination, history taking, team management, counseling, and communication skills. Most had experience with part-task trainers and standardized patients, but training with high fidelity mannequins and screen-based simulation was less common. Students’ expected SBME class size was six students, but instructors reported an average of 15.4. They also expected to have three hours per week of SBME. Most strongly agreed (Likert score 4.04–4.25) that SBME improves clinical practice, motivation, perceived safety, and performance. However, their scores were lower (3.85–3.95) with regard to their ability to utilize their knowledge and skills during SBME training. Medical students expected to undergo more SBME in 14 of 18 subjects (Anesthesiology, Biochemistry, Emergency medicine, Internal medicine, Obstetrics and Gynecology, Ophthalmology, Orthopedics, Otorhinolaryngology, Parasitology, Pathology, Pediatrics, Pharmacology, Physiology, and Psychiatry) than that they experienced with statistical significance (*p* < 0.05).

**Table 3:** SBME objectives and implementation according to Thai medical students (*N* = 367)

Topic				
**Learning objective (*n* (%))**	Clinical procedure			347 (94.6)
	Physical examination			346 (94.3)
	History taking			321 (87.5)
	Team management			299 (81.5)
	Counseling			273 (74.4)
	Communication skills			270 (73.6)
**Type of simulator (*n* (%))**	Part task trainer			355 (96.7)
	Standardized patients			331 (90.2)
	Cadaver			255 (69.5)
	High fidelity mannequin			200 (54.5)
	Screen-based simulation			60 (16.3)
**Expected parameters (mean (SD))**	Expected students per class (people)			6.0 (2.2)
	Expected duration per week (hours)			3.0 (2.2)
**Attitude **^**a**^** (mean (SD))**				
Experience with the simulation benefits clinical practice				4.23 (0.63)
Use of simulation increased my motivation to learn				4.15 (0.66)
I felt safe during the simulation				4.14 (0.73)
I understood the learning goal during the simulation				4.11 (0.72)
I was able to utilize prior knowledge during the simulation				3.85 (0.71)
I was able to utilize prior skills during the simulation				3.93 (0.72)
I was able to evaluate my performance in the simulation				4.22 (0.68)
Simulation-based training improves my teamwork skills				4.20 (0.72)
Simulation-based training improves my communication skills				4.04 (0.74)
Simulation-based training improves my clinical skills and competence				4.23 (0.61)
Simulation-based training improves my critical thinking and decision-making skills				4.25 (0.65)
**Subject (*n* (%))**		**Experience**	**Expected**	**Different in proportion (95%CI)**
Anatomy		248 (67.6)	244 (66.5)	1.0 (-5.8, 7.8)
Anesthesiology		273 (74.4)	299 (81.5)	7.1 (1.1, 13.1) ^b^
Biochemistry		43 (11.7)	70 (19.1)	7.4 (2.2, 12.6) ^b^
Community medicine		97 (26.4)	114 (31.1)	4.7 (-1.8, 11.2)
Emergency medicine		295 (80.4)	339 (92.4)	12.0 (7.0, 17.0) ^b^
Internal medicine		324 (88.3)	343 (93.5)	5.2 (1.0, 9.4) ^b^
Microbiology		55 (15.0)	74 (20.2)	5.2 (-0.3, 10.7)
Obstetrics and Gynecology		307 (83.7)	328 (89.4)	5.7 (0.8, 10.6) ^b^
Ophthalmology		209 (56.9)	264 (71.9)	15.0 (8.1, 21.9) ^b^
Orthopedics		243 (66.2)	298 (81.2)	15.0 (8.6, 21.4) ^b^
Otorhinolaryngology		214 (58.3)	264 (71.9)	13.6 (6.7, 20.5) ^b^
Parasitology		48 (13.1)	81 (22.2)	9.1 (3.6, 14.6) ^b^
Pathology		69 (18.8)	98 (26.7)	7.9 (1.8, 14.0) ^b^
Pediatrics		295 (80.4)	328 (89.4)	9.0 (3.8, 14.2) ^b^
Pharmacology		30 (8.2)	85 (23.2)	15.0 (9.7, 20.3) ^b^
Physiology		5 (1.4)	157 (42.8)	41.4 (35.4, 47.4) ^b^
Psychiatry		137 (37.3)	221 (60.2)	22.9 (15.7, 30.1) ^b^
Surgery		330 (89.9)	338 (92.1)	2.2 (-1.9, 6.3)

^a^Likert score: 1 = strongly disagree; 2 = disagree; 3 = neither agree nor disagree; 4 = agree; 5 = strongly agree

^b^Statistically significant

Table 4 shows data regarding SBME and essential procedural skills among Thai medical students. Percentages of students trained in procedural skills using SBME before practicing on actual patients varied between 41.7 to 99.5%. Eighteen procedures showed above 80% of students learning with SBME before practicing, and 12 procedures showed between 70–80% of students' experience with SBME. At the same time, 17 procedures showed that less than 70% of students had prior experience with SBME. Emergency care procedures (advanced life support, basic life support, endotracheal intubation, anterior nasal packing, venipuncture, and first aid management of injured patients) and obstetric and gynecologic procedures (normal labor and pelvic examination) showed high prior experience with SBME and a high satisfaction score. At the same time, non-emergency procedures were less used with SBME before practicing on patients. Some procedures showed less experience with SBME; however, students could practice on patients without harm, such as breathing exercises, oxygen therapy, aerosol bronchodilator therapy, and measurement of central venous pressure. We found that some procedures that should be trained using SBME before practicing, such as stomal care, foreign body removal from the vagina, capillary puncture, arterial puncture, and umbilical vein catheterization, were experienced with SBME less than 70%. Satisfaction scores on SBME varied between the procedures from 3.95 to 4.59 on five point-Likert scores. Twenty and 27 procedures showed agreed (mean of Likert 3.41–4.2) and strongly agreed (mean of Likert score above 4.2) on satisfaction with SBME, respectively.

**Table 4:** SBME and essential procedural skills among Thai medical students order by experience with SBME (*N* = 367)

Essential procedural skills	Experience with SBME (*n* (%))	Satisfaction score* (mean (SD))	Essential procedural skills	Experience with SBME (*n* (%))	Satisfaction score* (mean (SD))
Advanced life support	365 (99.5)	4.59 (0.73)	Insertion/removal of an intrauterine device	275 (74.9)	4.12 (1.01)
Basic life support	365 (99.5)	4.52 (0.76)	Marsupialization of Bartholin’s cyst	275 (74.9)	4.09 (1.04)
Endotracheal intubation	355 (96.7)	4.45 (0.85)	Vaginal packing	272 (74.1)	4.20 (0.92)
Suture	350 (95.4)	4.53 (0.76)	Aspiration of skin/bursa of elbow/ankle	262 (71.4)	4.31 (0.88)
Anterior nasal packing	348 (94.8)	4.34 (0.86)	Intramuscular injection	262 (71.4)	4.18 (0.92)
Urethral catheterization	340 (92.6)	4.49 (0.76)	Strengthening and stretching exercises	260 (70.8)	4.17 (0.92)
Normal labor	340 (92.6)	4.27 (0.90)	Intravenous injection	255 (69.5)	4.19 (0.94)
Incision/drainage of subcutaneous tissue	338 (92.4)	4.32 (0.89)	Cervical polypectomy	248 (67.6)	4.20 (0.95)
Venipuncture	335 (91.3)	4.08 (1.04)	Amniotomy	248 (67.6)	4.00 (1.04)
First aid management of injured patients	333 (90.7)	4.45 (0.74)	Cervical biopsy	241 (65.7)	4.19 (0.93)
Episiotomy and perineorrhaphy	333 (90.7)	4.21 (0.94)	Biopsy of skin and subcutaneous tissue	241 (65.7)	4.19 (0.91)
Pelvic examination	333 (90.7)	4.21 (0.98)	Debridement of wound	236 (64.3)	4.23 (0.98)
Papanicolaou smear	323 (88.0)	4.29 (0.85)	Oxygen therapy	233 (63.5)	4.34 (0.84)
Wound dressing	316 (86.1)	4.45 (0.82)	Aerosol bronchodilator therapy	226 (61.6)	4.10 (0.81)
Lumbar puncture	309 (84.2)	4.37 (0.94)	Capillary puncture	226 (61.6)	3.95 (1.06)
Stump bandaging	299 (81.2)	4.33 (0.87)	Breathing exercises	224 (61.0)	4.13 (0.96)
External splinting	297 (80.9)	4.39 (0.82)	Arterial puncture	221 (60.2)	4.21 (0.96)
NG tube irrigation and lavage	297 (80.9)	4.19 (0.94)	Umbilical vein catheterization	214 (58.3)	4.18 (0.97)
Local infiltration and digital nerve block	282 (76.8)	4.29 (0.83)	Measurement of central venous pressure	209 (56.9)	4.24 (0.90)
Excision of subcutaneous tissue cyst	280 (76.3)	4.25 (0.97)	Stomal care	209 (56.9)	4.19 (0.87)
Intravenous fluid infusion	280 (76.3)	4.09 (0.95)	Foreign body removal from the vagina	204 (55.6)	4.08 (0.98)
Subcutaneous injection	277 (75.5)	4.26 (0.88)	Blood and blood component transfusion	185 (50.4)	4.01 (0.97)
Skin traction of limbs	275 (74.9)	4.24 (0.86)	Phototherapy	153 (41.7)	3.98 (1.05)
Intradermal injection	275 (74.9)	4.19 (0.94)			

^*^Likert score: 1 = strongly disagree; 2 = disagree; 3 = neither agree nor disagree; 4 = agree; 5 = strongly agree

## Discussion

We found that SBME was commonly used in Thai medical schools but was mainly used to teach and evaluate psychomotor tasks, medical knowledge, patient care, and communication skills. However, it was less used for professionalism and leadership training, including research. The most common expected outcome of SBME was to improve performance and practice. Instructors mainly used part-task trainers, high-fidelity mannequins, and standardized patients. Administrators and instructors reported issues with the administration systems, faculty shortages, insufficient time and space, and a lack of simulators. Students reported having experienced SBME to achieve various learning objectives using several types of simulators. However, they had expected fewer students per class than the number of students per class reported by instructors, and they expected at least three hours of SBME per week. Students strongly agreed that SBME improved self-evaluation, skills, critical thinking, teamwork, essential procedural skills, and decision-making but less so that it improved perceived safety and communication or allowed them to utilize their knowledge and skills.

We found that most schools used SBME for teaching and evaluation, but few employed SBME in research. The simulation could be used as a research tool to test and improve patient safety and efficiency. A 2010 study by the American Medical College in North American medical schools reported an overall SMBE usage rate of 86% in education, 71% in assessment, and 40% in research [7, 8]. Our data regarding education and assessment were comparable, but we found less usage in research. This suggests that a greater focus on SBME in research may be required. Our finding that SBME is primarily used in Thailand to develop knowledge and technical and non-technical skills is similar to the findings of a previous study in low and middle-income countries (IMICs), which found that SBME was mainly used to teach medical expertise and communication skills [6]. However, that study reported the most common expected outcomes of SBME to be improvements in knowledge, followed by performance, and patient outcome, while the most- to least-common expected outcomes in our study were improvements in performance, practice, knowledge, attitude, and patient outcomes. The most common simulators used in our study and the previously mentioned study in IMICs were part-task trainers, standardized patients, and high-fidelity mannequins. However, our respondents reported less immersive-environment and in situ simulation and practice in actual clinical environments than in high-income countries that aim to prepare medical students for practice as junior doctors [6, 10].

Reported obstacles included issues with administration systems and insufficient faculty, space, simulators, and financial support. Previous studies in IMICs in Southeast Asia and elsewhere have reported similar obstacles. For example, a 2005 medical education study in Southeast Asia (including Thailand) cited financial difficulties, lack of faculty, and faculty resistance to change as challenges in implementing SBME [11]. These sorts of obstacles may be the reason SBME is not a favored educational technique among instructors in Thailand. Another study of SBME in IMICs found low rates of SBME use among instructors and that most institutions used low-tech simulators and focused on knowledge and skill outcomes. More robust employment of SBME should be encouraged in order to improve quality of care and patient safety [5, 6]. SBME could be the research tool that aims to understand the problem situation, assess intervention feasibility, evaluate the effect of the intervention, evaluate how the intervention is received by participants, and feed data to economic models [5]. A study of SBME in Japan reported that a larger SBME faculty was associated with greater SBME implementation at the resident level [12]. Previous studies in other countries have reported an average of 13 personnel per simulation training center [5, 6]. However, our participants reported shortages of simulation personnel and other supportive staff, which likely has a direct impact on SBME in Thailand. Furthermore, many medical schools we surveyed did not have a position for simulation specialists, simulation technicians, educators, and researchers in the simulation center. This presents a significant development opportunity, as these faculty members may be a critical factor in determining the success or failure of SBME [13]. Half of the instructors we surveyed reported insufficient faculty training, which is consistent with the findings of another study in IMICs that only 70% of instructors had received formal training in SBME and that training duration varied from half a day to multiple days, though it was most often less than one week [6]. Lack of time, training, and incentives has been reported as commonly cited barriers to faculty-related improvements [14]. Insufficient time can often lead to inadequate implementation of SBME due to the competing demands of other types of instruction, and faculty often find it challenging to carve out sufficient time to apply SBME in their teaching. It is necessary that instructors’ workloads are adjusted as appropriate, as SBME requires a significant amount of time to prepare to teach. Mitigating the effects of these obstacles might lead to increased SBME usage in Thailand.

Medical students in our study responded that they expected to experience more SBME in some subjects. They also indicated a desire for fewer students per class and more hours of SBME per week. Teaching strategies should be improved to respond to these expectations. We also found that essential procedural skill training using SBME benefits the learners and can positively affect patient safety and quality of care. However, more training and improved teaching methods are currently required. In a previous study, instructors, junior doctors, and medical students indicated that priority should be placed on practical resource management skills in order to narrow the gap between expected and observed competency [15].

Participants in our study expressed positive views regarding SBME. This is consistent with a previous study in Southeast Asia, which found strong administrative support to be a strength of SBME in Thailand [11]. Another study found that training that was goal-oriented, self-directed, individual, outcomes-based rather than time-based, and included deliberate practice was critical in improving students’ attitudes toward SBME in Thailand [16].

One limitation of this study was selection bias, as those interested in SBME were more likely to participate in the survey, thus affecting the results. Although we received a satisfying response rate among administrators, those of instructors and medical students were not as high. The low response rate among instructors may have been due to the low total number of instructors using SBME. As we distributed the instructor’s questionnaire through a contact person in each medical school, who would distribute the survey within their institution, we were not able to predict the response rate. Another limitation was that we only gathered information regarding overall SBME implementation but did not explore specific cases in detail in order to reduce the amount of time required to fill out the questionnaire. Despite these limitations however, our study provided valuable data regarding the impact of SBME in Thailand.

## Conclusions

Our results showed that the focus of SBME was mainly limited to teaching and assessment and that effective implementation would require more faculty, space, simulators, and financial resources. In addition, students’ expectations regarding SBME are not currently being met and need to be addressed. Despite these challenges, the administrators, instructors, and medical students surveyed expressed positive attitudes toward SBME.

## Disclosures

Not known.

## Supplementary Information

**Additional file 1. **Questionnaires (administrator, instructor, and medical student).

## Data Availability

The datasets generated during and analyzed during the current study are not publicly available due to the confidentiality announcement made on the participants but are available from the corresponding author upon reasonable request.
