# Supplementary material for: Simulation-based medical education in Thailand: a cross-sectional online national survey
Source: BMC Med Educ. 2022 Apr 20;22:298. doi: 10.1186/s12909-022-03369-9 (PMC9019967; doi:10.1186/s12909-022-03369-9)
Supplement: Supplementary file 1 — Additional file 1. Questionnaires (administrator, instructor, and medical student). [file 12909_2022_3369_MOESM1_ESM.pdf]

Administrator questionnaire

Simulation-based medical education (SBME): A technique to replace, augment or amplify reality with guided experiences, often immersive, that evoke or replicate substantial aspects of the real world in an interactive fashion.

Consent form

Participant characteristics

- Name [ ]
- Position [ ]
- Medical school name [ ]
- Phone number [ ]
- E-mail [ ]

SBME implementation and objectives

- SBME is used in my school [ ] yes [ ] no (end the questionnaire)
- Communication skills [ ] yes [ ] teaching [ ] evaluation/feedback/certification [ ] research [ ] no
- Decision making [ ] yes [ ] teaching [ ] evaluation/feedback/certification [ ] research [ ] no
- Leadership [ ] yes [ ] teaching [ ] evaluation/feedback/certification [ ] research [ ] no
- Medical knowledge [ ] yes [ ] teaching [ ] evaluation/feedback/certification [ ] research [ ] no
- Patient care [ ] yes [ ] teaching [ ] evaluation/feedback/certification [ ] research [ ] no
- Professionalism [ ] yes [ ] teaching [ ] evaluation/feedback/certification [ ] research [ ] no
- Psychomotor tasks [ ] yes [ ] teaching [ ] evaluation/feedback/certification [ ] research [ ] no
- Team management [ ] yes [ ] teaching [ ] evaluation/feedback/certification [ ] research [ ] no

SBME obstacles

- Administration system [ ] Insufficient financial support [ ] No administration system [ ] No simulation center [ ] none of above
- Space shortages [ ] Controlled room [ ] Debrief room [ ] Lecture room [ ] Observation room [ ] Office room [ ] Storage room [ ] Training room [ ] none of above
- Simulators shortages [ ] Cadaver [ ] High fidelity mannequin [ ] Part task trainer [ ] Screen-based simulation [ ] Standardized patients [ ] none of above
- Faculty shortages [ ] Course director [ ] Educator [ ] Instructors [ ] Officer [ ] Researcher [ ] Researcher assistant [ ] Simulation technician [ ] Standardized patient trainer [ ] none of above

Attitude regarding SBME

Likert score: 1=strongly disagree; 2=disagree; 3=neither agree nor disagree; 4=agree; 5=strongly agree

- SBME is essential to the medical curriculum [ ]
- Integration of SBME into the medical curriculum is necessary [ ]
- SBME should be encouraged in medical schools [ ]

Instructor questionnaire

Simulation-based medical education (SBME): A technique to replace, augment or amplify reality with guided experiences, often immersive, that evoke or replicate substantial aspects of the real world in an interactive fashion.

Consent form

Participant characteristics

- E-mail [ ]
- Age [ ]
- Medical school name [ ]
- Department [ ]

SBME implementation and objectives

- SBME is used in my course(s) [ ] yes [ ] no (end the questionnaire)

Any courses for 1st-3rd year medical students

- The expected outcomes of SBME (≥ 1)
  [ ] Improving knowledge [ ] Improving attitude [ ] Improving performance [ ] Improving practice
  [ ] Change patient outcome
- Types of simulators (≥ 1)
  [ ] Cadaver [ ] High fidelity mannequin [ ] Part task trainer [ ] Screen-based simulation
  [ ] Standardized patients
- Teaching characteristics (on average)
  - Teaching duration (hours) [ ]
  - Preparation duration (hours) [ ]
  - Courses per year (number) [ ]
  - Students per class (person) [ ]

Any courses for 4th-6th year medical students

- The expected outcomes of SBME (≥ 1)
  [ ] Improving knowledge [ ] Improving attitude [ ] Improving performance [ ] Improving practice
  [ ] Change patient outcome
- Type of simulators (≥ 1)
  [ ] Cadaver [ ] High fidelity mannequin [ ] Part task trainer [ ] Screen-based simulation
  [ ] Standardized patients
- Teaching characteristics (on average)
  - Teaching duration (hours) [ ]
  - Preparation duration (hours) [ ]
  - Courses per year (number) [ ]
  - Students per class (people) [ ]

SBME obstacles

- faculty [ ] inadequate [ ] adequate [ ] excessive [ ] no data
- faculty time [ ] inadequate [ ] adequate [ ] excessive [ ] no data
- faculty training [ ] inadequate [ ] adequate [ ] excessive [ ] no data
- financial support [ ] inadequate [ ] adequate [ ] excessive [ ] no data
- available space [ ] inadequate [ ] adequate [ ] excessive [ ] no data
- simulators [ ] inadequate [ ] adequate [ ] excessive [ ] no data

Medical student questionnaire

Simulation-based medical education (SBME): A technique to replace, augment or amplify reality with guided experiences, often immersive, that evoke or replicate substantial aspects of the real world in an interactive fashion.

Consent form

Participant characteristics

- Age [ ]
- Gender [ ]
- Medical school name [ ]

SBME objectives and implementation

- SBME experience [ ] yes [ ] no (end the questionnaire)
- Learning objectives ($\geq$ 1) [ ] Clinical procedure [ ] Communication skills [ ] Counseling [ ] History taking
  [ ] Physical examination [ ] Team management
- Types of simulators ($\geq$ 1) [ ] Cadaver [ ] High fidelity mannequin [ ] Part task trainer
  [ ] Screen-based simulation [ ] Standardized patients
- Experience and expectations regarding SMBE by subject
    - Anatomy experience [ ] yes [ ] no; expected SBME [ ] yes [ ] no
    - Anesthesiology experience [ ] yes [ ] no; expected SBME [ ] yes [ ] no
    - Biochemistry experience [ ] yes [ ] no; expected SBME [ ] yes [ ] no
    - Community medicine experience [ ] yes [ ] no; expected SBME [ ] yes [ ] no
    - Emergency medicine experience [ ] yes [ ] no; expected SBME [ ] yes [ ] no
    - Internal medicine experience [ ] yes [ ] no; expected SBME [ ] yes [ ] no
    - Microbiology experience [ ] yes [ ] no; expected SBME [ ] yes [ ] no
    - Parasitology experience [ ] yes [ ] no; expected SBME [ ] yes [ ] no
    - Pathology experience [ ] yes [ ] no; expected SBME [ ] yes [ ] no
    - Pediatrics experience [ ] yes [ ] no; expected SBME [ ] yes [ ] no
    - Pharmacology experience [ ] yes [ ] no; expected SBME [ ] yes [ ] no
    - Physiology experience [ ] yes [ ] no; expected SBME [ ] yes [ ] no
    - Psychiatry experience [ ] yes [ ] no; expected SBME [ ] yes [ ] no
    - Obstetrics and gynecology
      experience [ ] yes [ ] no; expected SBME [ ] yes [ ] no
    - Ophthalmology experience [ ] yes [ ] no; expected SBME [ ] yes [ ] no
    - Orthopedics experience [ ] yes [ ] no; expected SBME [ ] yes [ ] no
    - Otorhinolaryngology experience [ ] yes [ ] no; expected SBME [ ] yes [ ] no
    - Surgery experience [ ] yes [ ] no; expected SBME [ ] yes [ ] no
- Expected SBME implementation
    - Expected students per class (people) [ ]
    - Expected duration per week (hours) [ ]
- Essential procedural skills
  (Likert score: 1=strongly disagree; 2=disagree; 3=neither agree nor disagree; 4=agree; 5=strongly agree)
    - Advanced life support experience before practice [ ] yes [ ] no ; satisfaction scale [ ]
    - Aerosol bronchodilator therapyexperience before practice [ ] yes [ ] no ; satisfaction scale [ ]
    - Amniotomy experience before practice [ ] yes [ ] no ; satisfaction scale [ ]
    - Anterior nasal packing experience before practice [ ] yes [ ] no ; satisfaction scale [ ]
    - Arterial puncture experience before practice [ ] yes [ ] no ; satisfaction scale [ ]
    - Aspiration of skin/bursa of elbow/ankle
      experience before practice [ ] yes [ ] no ; satisfaction scale [ ]
    - Basic life support experience before practice [ ] yes [ ] no ; satisfaction scale [ ]
    - Biopsy of skin and subcutaneous tissue
      experience before practice [ ] yes [ ] no ; satisfaction scale [ ]
    - Blood and blood component transfusion
      experience before practice [ ] yes [ ] no ; satisfaction scale [ ]
    - Breathing exercises experience before practice [ ] yes [ ] no ; satisfaction scale [ ]
    - Capillary puncture experience before practice [ ] yes [ ] no ; satisfaction scale [ ]
    - Cervical cervix biopsy experience before practice [ ] yes [ ] no ; satisfaction scale [ ]

- Cervical cervix polypectomy experience before practice [ ] yes [ ] no ; satisfaction scale [ ]
- Wound Debridement experience before practice [ ] yes [ ] no ; satisfaction scale [ ]
- Endotracheal intubation experience before practice [ ] yes [ ] no ; satisfaction scale [ ]
- Episiotomy and perineorrhaphy
  experience before practice [ ] yes [ ] no ; satisfaction scale [ ]
- Excision of subcutaneous tissue cyst
  experience before practice [ ] yes [ ] no ; satisfaction scale [ ]
- External splinting experience before practice [ ] yes [ ] no ; satisfaction scale [ ]
- First aid management of an injured patient
  experience before practice [ ] yes [ ] no ; satisfaction scale [ ]
- Foreign body removal from the vagina
  experience before practice [ ] yes [ ] no ; satisfaction scale [ ]
- Incision/drainage of subcutaneous tissue
  experience before practice [ ] yes [ ] no ; satisfaction scale [ ]
- Insertion/removal of intrauterine device
  experience before practice [ ] yes [ ] no ; satisfaction scale [ ]
- Intradermal injection experience before practice [ ] yes [ ] no ; satisfaction scale [ ]
- Intramuscular injection experience before practice [ ] yes [ ] no ; satisfaction scale [ ]
- Intravenous fluid infusion experience before practice [ ] yes [ ] no ; satisfaction scale [ ]
- Intravenous injection experience before practice [ ] yes [ ] no ; satisfaction scale [ ]
- Local infiltration and digital nerve block
  experience before practice [ ] yes [ ] no ; satisfaction scale [ ]
- Lumbar puncture experience before practice [ ] yes [ ] no ; satisfaction scale [ ]
- Marsupialization of Bartholin's cyst
  experience before practice [ ] yes [ ] no ; satisfaction scale [ ]
- Measurement of central venous pressure
  experience before practice [ ] yes [ ] no ; satisfaction scale [ ]
- NG tube irrigation and lavage experience before practice [ ] yes [ ] no ; satisfaction scale [ ]
- Normal labor experience before practice [ ] yes [ ] no ; satisfaction scale [ ]
- Oxygen therapy experience before practice [ ] yes [ ] no ; satisfaction scale [ ]
- Papanicolaou smear experience before practice [ ] yes [ ] no ; satisfaction scale [ ]
- Pelvic examination experience before practice [ ] yes [ ] no ; satisfaction scale [ ]
- Phototherapy experience before practice [ ] yes [ ] no ; satisfaction scale [ ]
- Skin traction of limbs experience before practice [ ] yes [ ] no ; satisfaction scale [ ]
- Stomal care experience before practice [ ] yes [ ] no ; satisfaction scale [ ]
- Strengthening and stretching exercises
  experience before practice [ ] yes [ ] no ; satisfaction scale [ ]
- Stump bandaging experience before practice [ ] yes [ ] no ; satisfaction scale [ ]
- Subcutaneous injection experience before practice [ ] yes [ ] no ; satisfaction scale [ ]
- Suture experience before practice [ ] yes [ ] no ; satisfaction scale [ ]
- Umbilical vein catheterization experience before practice [ ] yes [ ] no ; satisfaction scale [ ]
- Urethral catheterization experience before practice [ ] yes [ ] no ; satisfaction scale [ ]
- Vaginal packing experience before practice [ ] yes [ ] no ; satisfaction scale [ ]
- Venipuncture experience before practice [ ] yes [ ] no ; satisfaction scale [ ]
- Wound dressing experience before practice [ ] yes [ ] no ; satisfaction scale [ ]

- **Attitude toward SBME**
  (Likert score: 1=strongly disagree; 2=disagree; 3=neither agree nor disagree; 4=agree; 5=strongly agree)
  - Experience with simulation benefits clinical practice. [ ]
  - Use of simulation increased my motivation to learn. [ ]
  - I felt safe during simulation. [ ]
  - I understood the learning goal during simulation. [ ]
  - I was able to utilize prior knowledge during simulation. [ ]
  - I was able to utilize prior skills during simulation. [ ]
  - I was able to evaluate my performance in the simulation. [ ]
  - Simulation-based training improves my teamwork skills. [ ]
  - Simulation-based training improves my communication skills. [ ]
  - Simulation-based training improves my clinical skills and competence. [ ]
  - Simulation-based training improves my critical thinking and decision-making skills. [ ]
